# Mini-Review on Capacity-Building for Data-Driven Early Childhood Systems: The Consortium for Pre-primary Data and Measurement in Sub-Saharan Africa

**DOI:** 10.3389/fpubh.2020.595821

**Published:** 2021-02-25

**Authors:** Abbie Raikes, Rebecca Sayre, Dawn Davis

**Affiliations:** ^1^College of Public Health, University of Nebraska Medical Center, Omaha, NE, United States; ^2^ECD Measure, University of Nebraska Lincoln, Lincoln, NE, United States; ^3^University of Nebraska Lincoln, Lincoln, NE, United States

**Keywords:** early childhood, measurement, capacity-building, low- and lower-middle-income countries, data - driven learning

## Abstract

Low- and middle-income countries (LMIC) are increasing investments in early childhood development programs, including early childhood education. As programs reach scale, there is increasing demand for evidence on impacts of investments. Little work to date has examined capacity required to effectively use data at scale in LMIC, including opportunities and barriers to integrating data into ongoing program implementation and tracking child development and quality of services at scale. Below, we outline the rationale and approach of the Consortium for Pre-primary Data and Measurement in Sub-Saharan Africa, focused on building capacity for data-driven decision-making in early childhood systems. Themes from the first phase include the importance of building diverse groups of stakeholders to define priorities for data and measurement, the need for coordinated and strategic investments in data and measurement, and the value of long-term investments in government/civil society/university partnerships to generate locally relevant data on early childhood education.

## The Challenge Facing ECE: Scaling Quality Programs

Early childhood care and education (ECE) programs can be highly effective interventions, leading to improved outcomes for children throughout schooling and into adulthood (1, 2). Stimulating, supportive care in early childhood leads to substantial benefits for children (3), including quality ECE with responsive, interactive teachers, access to materials, and safe and stimulating environments received before children enter primary school. Many countries have increased investments in ECE (defined as formal preschools, community-based preschools, childcare settings, and parenting programs). The passage of the Sustainable Development Goals' target focused on early childhood care and education, Target 4.2, underscored the importance of early childhood for later learning and development and emphasized the value of measurement to track progress toward goals (4). Countries now face a critical challenge in ensuring access to quality early childhood programs for all children. The most vulnerable children are still the most likely to be excluded from high-quality early childhood services (3) and there are notable disparities in access to ECE based on family wealth and geography (5).

Providing children access to quality ECE is a challenge globally, but perhaps especially in low- and middle-income countries (LMIC) facing fewer resources available for ECE; a greater population of children facing considerable contextual risks; and a strong need to work across health, nutrition, social protection, and education sectors to address children's development holistically (3). Despite this complexity, the infrastructure for scaling may be lacking, leading to expansion of early childhood systems without well-developed monitoring systems to track quality of programs over time (6). Rapid expansions of access can lead to variable levels of quality within preschool settings, due to uneven training of teachers, limited access to materials, and the unique contextual challenges and opportunities of different locales (7). ECE may also be operating in the context of considerable urban/rural disparities, teacher shortages, and underfunded schools that create pressure on ECE classrooms to accommodate large numbers of children with few resources (8). These factors work together to create suboptimal conditions for quality ECE in many LMIC (9).

Despite the value of using evidence to inform decision-making, there are still few conclusions on how best to promote the use of evidence in education (10). Our review addresses available literature on using data to improve ECE with emphasis on two primary questions: first, what do we know about how to encourage data use to inform ECE policies and programs; and second, what practices may facilitate data use? We offer an example of capacity-building, the Consortium for Pre-primary Data and Measurement in Sub-Saharan Africa (CPDMA). We conclude with themes that emerged from the Consortium's first year and the implications for future efforts to build capacity for measurement, evaluation, and learning at scale in ECE.

### Role of Data and Measurement in Scaling

Research, measurement, and data play a central role in scaling early childhood programs, by tracking participation; quality of implementation; and impacts on children and families over time, especially whether goals for equity in access and learning are being achieved (4, 11). While several global reports on ECE have provided invaluable data on the status of young children (i.e., annual State of the World's Children; UNICEF; Global Education Monitoring Report, UNESCO), local evidence is critical for implementation. Country context has a strong impact on implementation of early childhood programs, and it is essential to define what works for whom, as the impact of early childhood programs varies by context and across children and families (12, 13). Moreover, careful attention to program implementation is necessary to achieve the promise of early childhood programs (14), including focus on the quality of ECE staff, provision of materials, clarity of curricular and programmatic goals, and ongoing support for professional development. Data systems are needed to reliably collect information on implementation and program impacts, and good systems can play a pivotal role in creating cohesive, effective intersectoral approaches to addressing disparities (15, 16), and fundamentally can contribute toward clarifying which children and families are benefiting from programs in various contexts (17).

However, the challenges in using data, measurement, and research to improve policies and practices are well-documented [e.g., (18, 19)]. For research to influence practice, for example, there must be tight linkages and relationships between the producers and users of research, and the organizations they represent (18), requiring substantial staff capacity to produce reliable, relevant results, and use the data to influence policies and practices. Resources for early childhood are limited in many LMIC, with few data to track implementation, which is especially problematic when scaling programs to serve large populations of children facing challenging contexts (20). Getting effective ECE monitoring systems in place requires considerable financial and human resources (6). Even when data are available, many of the recurring surveys are high-level and intended for policy, providing little direct feedback for teachers and other early childhood professionals to improve their practices.

In sum, early childhood data are important for quality ECE and should include data on implementation and outcomes with emphasis on the relationships needed to ensure data are used. Given these insights, how should we approach capacity-building for using research, data, and measurement to improve programs and policies, especially in LMIC, and how can we collectively help build that capacity?

### Building Capacity for Data Use in ECE

#### Consortium for Pre-primary Data and Measurement

The CPDMA was initiated in 2018 by USAID and the ECD Measure Group at the University of Nebraska Medical Center to build the capacity to generate and use ECE data in sub-Saharan Africa. As part of the 2017 P.L. 115-56, the Reinforcing Education Accountability in Development (READ) Act^1^, Congress called for a comprehensive international education strategy, which included a new focus on the role of pre-primary education. As a response to the READ Act, the 2018 USAID Education Policy added pre-primary education to USAID's priority areas, recognizing that ECE lays the foundation to long-term student learning outcomes. One of the main principles within the policy is using evidence and data to drive decision-making and USAID investments. Under these new emphases, USAID and ECD Measure identified the need to build pre-primary data and measurement capacity at the country level.

In its first phase from 2018 to 2020, CPDMA supported countries in identifying their ECE needs and facilitated cross-country learning to build on best practices from the continent. Four country task force teams were formed in partnership with USAID country missions in Ethiopia, Liberia, Rwanda, and South Africa. Because ECE-related data and measurement require working across partners and organizations, these multi-disciplinary task force teams comprised government officials, local university researchers, ECE practitioners from civil society, and USAID education officers. Country teams engage in country-level planning and dialogue as well as participate in virtual and in-person cross-country meetings.

#### CPDMA Guiding Principles

Based on experiences with previous measurement projects [i.e., Measuring Early Learning & Outcomes; see (21), for a review], a set of key principles were articulated to guide the project. First, previous work had documented that countries valued data to inform national planning (22), so CPDMA prioritized country-driven decision-making on where data were needed, and thus left the scope open-ended to allow country teams to define their priorities. Second, the goal of our work was to contribute to the local capacity that would be required to conceptualize, implement, and sustain high-value data work. A key marker of success in the project's first phase was the functioning of the country teams and their ability to work together to identify high-leverage data and measurement work.

#### CPDMA Project Activities

**Figure 1:**
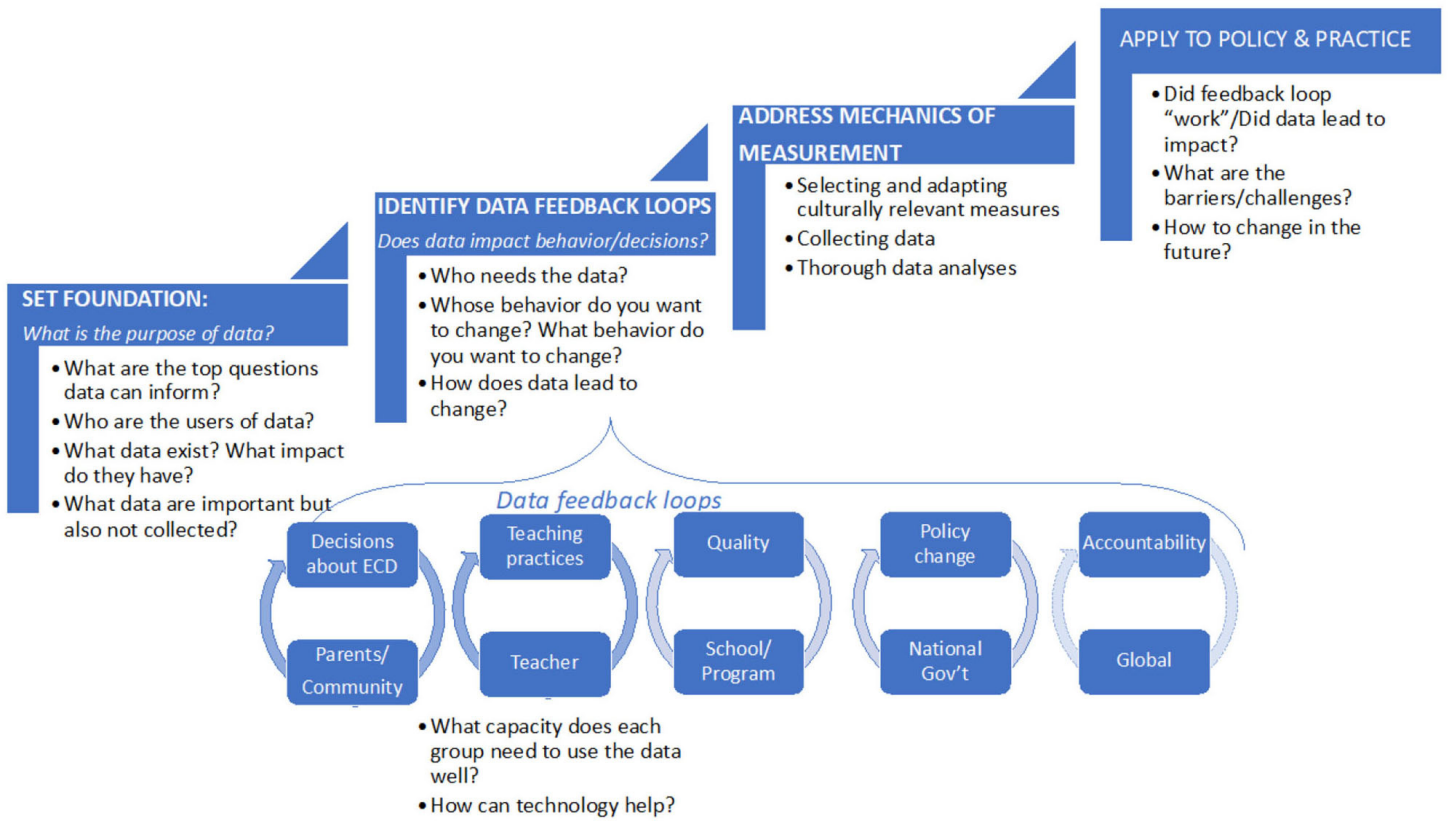
Data for impact framework.

As a starting point for the country team's work, a *Data for Impact* framework and toolkit was prepared based on existing literature and experiences with policy and program development to guide country teams through the process of building data-driven ECE systems (23). The framework proposes four essential steps: (1) identify the purposes of ECE data, including places where data may have impact; (2) define data feedback loops; (3) address the mechanics of measurement; and (4) apply to policy and practice (see Figure 1). These steps are explained below.

**Figure 2:**
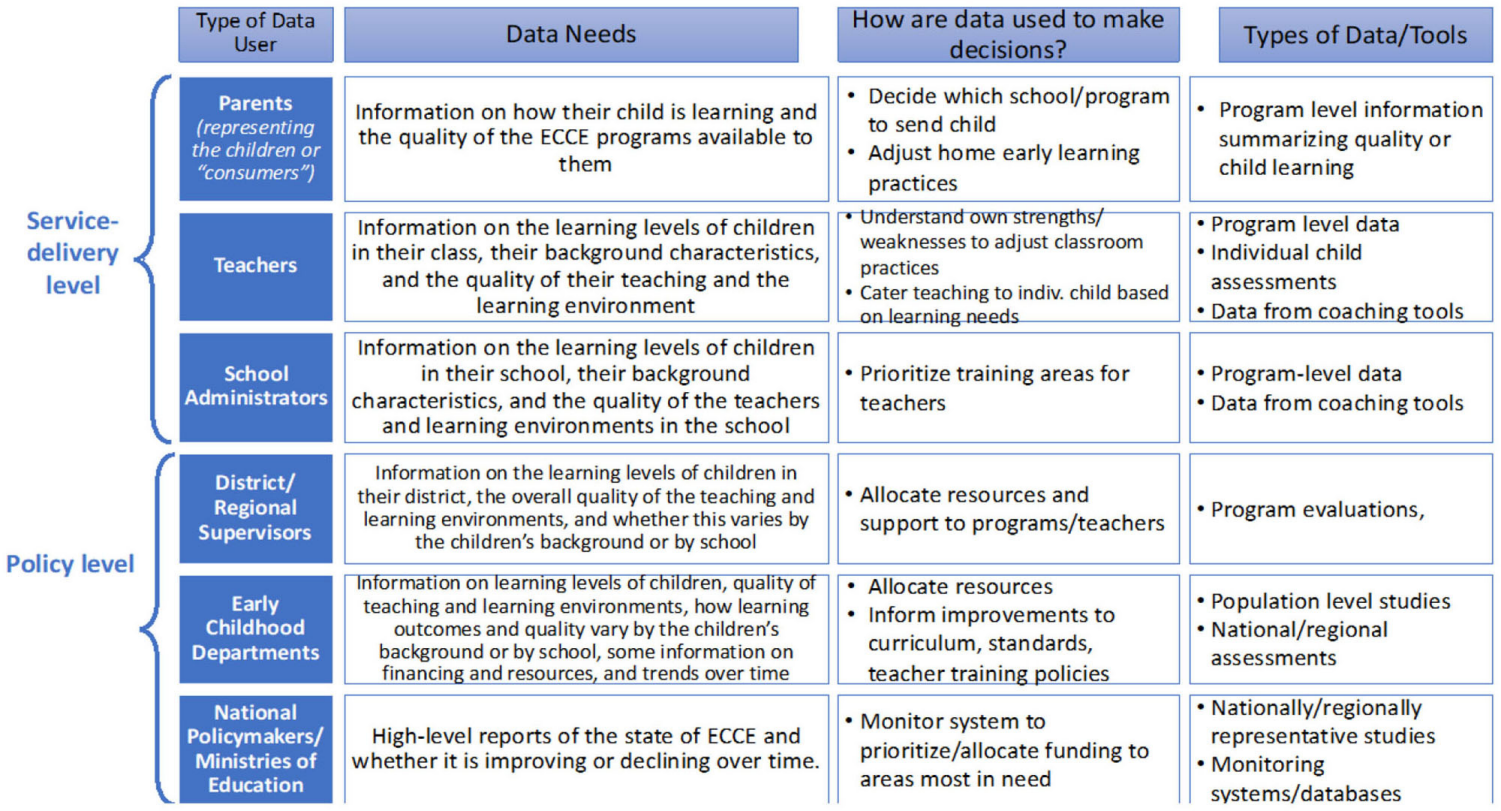
ECE data users and data needs.

During the task force's inception year, country teams composed of government, civil society, and university representatives focused on the first two steps of the framework. First, country teams conducted a diagnostic data mapping exercise to outline priorities for ECE data in their respective countries. Figure 2 illustrates the types of data users and data needs created to guide country teams. Teams defined the purpose of ECE data by considering locally relevant questions on ECE programs and policies, as well as the audience for data in an ECE system (parents, teachers, administrators, policymakers, etc.) (see Figure 2). Teams then identified existing ECE data and how these data are used [for example, data on ECE from Education Management Information System (EMIS), program evaluations conducted by civil society organizations, and research studies]. This step revealed several opportunities to use existing data to address questions identified in the first step. However, these data are not always available (or known) to the ECE community, and teams concluded that better collaboration could improve the effectiveness and efficiency of using ECE-related data in the future.

As a second step, teams identified “feedback loops” for data, to help prioritize which gaps to fill and started planning possible projects to build toward a more data-driven ECE system. To articulate feedback loops in each country context, teams outlined where data were or could be used to influence policy and practice in ECE. Teams considered the types of decisions users of data make or will be making, for example, how teachers might use data to monitor student progress, and how administrators would use data to monitor ECE access. The relationships that are required to promote data use were also considered. Establishing tight feedback loops between policy and practice requires regular sharing of information and ongoing implementation data in a trusting and transparent manner (24), beginning with specific programs to refine methods for using data and expanding over time.

Country teams will address the two final steps in future work. The third step, mechanics of measurement, refers to the tools and processes required for reliable data, such as which measurement tools to use. The fourth step, applying to policy and practice, refers to the implementation of the feedback loops identified in the second step; for example, sharing results with ECE stakeholders or using data to help teachers identify best practices in ECE classrooms.

#### Examples of Data-Driven Early Childhood Projects

Throughout the course of the project, several examples of data-driven projects were identified. A CPDMA member from South Africa, Grow Early Child Development^2^, has created effective data feedback loops at the service-delivery level, by providing childcare providers with information on markers of quality and routinely using technology-enabled data collection to track program performance. Grow ECD is a franchise of South African ECE Centers serving disadvantaged communities and uses a data-driven approach to ensure that ECE facilities meet service delivery standards. The organization has recently developed an app that provides an opportunity for ongoing feedback loops, as stakeholders interact with real-time data from their ECE centers and classrooms.

Rwanda's National Early Childhood Development Program (NECDP)^3^ has been regionally recognized for promoting a national intersectoral data system. The multi-sectoral work of NECDP requires an integrated data system that reflects a coordinated approach to early childhood development, cutting across health, nutrition, social protection, and ECE. NECDP has recently established an ECE dashboard to hold different public institutions accountable for their work. Seven national ministries or agencies contribute ECD data to the dashboard, each using distinct indicators aligned with its own sectoral priorities. The dashboard promotes a data feedback loop at the national level, where national line ministries and agencies report and hold each other accountable on their respective sectoral progress in ECE programming.

## Discussion On Building Local Capacity for Data and Measurement

Several themes emerged from CPDMA's work that have implications for future capacity-building efforts in early childhood.

First, CPDMA emphasized the value of diverse stakeholder groups coalesced around early childhood data. In several situations, country task force members were not aware of data and measurement work in other organizations within their own countries. Engagement with a diverse set of stakeholders at the country level contributed to bridging gaps between organizations working on ECE data and measurement in policy, research, and practice. Including ministerial counterparts with direct connections to policy decisions has ensured that each country team's efforts aligned with existing national priorities. The involvement of local researchers and implementers enriched the interdisciplinary dialogue for each country team. The CPDMA task force also created a new opportunity for focused dialogue on ECE measurement. Country teams expressed that CPDMA's convening facilitated having regular country team meetings, which provided accountability that helped keep momentum among in-country actors. The inter-disciplinary and multi-sectoral composition of each country team also allowed for multiple perspectives in discussion on data and measurement.

Teams also appreciated the opportunity to network with peers within the sub-Saharan Africa region. Members expressed that the cross-country CPDMA task force contributed to the motivation, interest, and confidence necessary to address data and measurement within early childhood settings. CPDMA provided an opportunity for country teams to learn from examples from other countries and think through the mechanisms by which data in ECE can lead to improvement. Yet these stakeholder groups face many of the same issues faced by intersectoral efforts in early childhood more broadly—namely, the challenges of working across multiple agencies with distinct, sometimes complementary and sometimes contradictory goals. The creation of stakeholder groups was one initial step toward creating stronger infrastructure for scaling; data systems are an essential piece of this process and can serve as a driver for positive change (12), but only with concerted effort.

Second, coordinated, focused data may lead to greater leverage for policy and programmatic investments. During CPDMA, countries identified multiple sources of ECE data. However, data were often not coordinated, were broad in scope, may not have included all populations of children, and had varying degrees of connection policy priorities, potentially lessening the impact of data. There were sometimes few data focused on the quality of services, despite the importance of monitoring program quality and improvement. For the CPDMA task force country teams, the examples demonstrating how data can leverage change in ECE—at the provider, classroom/school, and national levels—were helpful in further delineating the long-term goal of investing in ECE data. One way to help achieve this goal is to create a shared data dashboard for all sectors, such as the NECDP dashboard in Rwanda, or to use ongoing data collection to drive professional development, as in the Grow ECD model.

Third, attention should be placed on monitoring quality of programs, in addition to measuring child development and learning. Many teams were especially interested in determining whether ECE programs were successful in supporting children's development. Yet, to implement highly effective programs, investments must be made in using data to guide professional development for ECE teachers, sharing information with program staff and key partners who support programs, and applying findings to program improvement efforts (25). One U.S. example using this model is the Educare Learning Network^4^ The Educare Learning Network is a consortium of early childhood education programs serving at-risk children and families that utilizes research-program partnerships to implement a set of core features including data utilization to inform embedded professional development and support for high-quality teaching practices and family engagement that leads to long-term positive child and family outcomes [see (26), for an overview; (27, 28)].

Although there is growing interest in improving services for young children, many countries will likely see continued resource constraints for early childhood programs. To provide countries with the data necessary to track the quality and reach of early childhood investments, it will be necessary to devise more efficient, faster, and more actionable data on early childhood. This work adds a concrete example of capacity-building on data use in ECE. Our findings suggest that future efforts should focus on engaging a broad range of stakeholders, defining clear pathways by which data can influence ECE, and documenting examples of using data at multiple levels of the ECE system. More research is needed in the future to systematically evaluate the impact of data on decision-making in ECE, how to build systems that promote coordinated and ongoing monitoring and support for quality programming across sectors, how to engage teachers and parents in ECE data, and how to efficiently build the infrastructure required to generate actionable, reliable data over time. The many demands on early childhood systems can relegate data and measurement to a back seat—yet the critical role of data systems for monitoring implementation and impacts on children and families should not be forgotten.

## Author Contributions

AR took the lead in conceptualizing and drafting the paper. RS wrote about the details of the Consortium's work. DD was a technical contributor to the project. She described the project's conclusions and contributed to the description of the work of the Consortium.

## Conflict of Interest

The authors declare that the research was conducted in the absence of any commercial or financial relationships that could be construed as a potential conflict of interest.

## Footnotes

^1^https://www.congress.gov/115/plaws/publ56/PLAW-115publ56.pdf

^2^https://www.growecd.org.za/

^3^http://ecd.gov.rw/

^4^https://www.educareschools.org/our-approach/educare-learning-network/
